# Urban Scaling and the Production Function for Cities

**DOI:** 10.1371/journal.pone.0058407

**Published:** 2013-03-27

**Authors:** José Lobo, Luís M. A. Bettencourt, Deborah Strumsky, Geoffrey B. West

**Affiliations:** 1 School of Sustainability, Arizona State University, Tempe, Arizona, United States of America; 2 Santa Fe Institute, Santa Fe, New Mexico, United States of America; 3 Department of Geography and Earth Sciences, University of North Carolina at Charlotte, Charlotte, North Carolina, United States of America; MIT, United States of America

## Abstract

The factors that account for the differences in the economic productivity of urban areas have remained difficult to measure and identify unambiguously. Here we show that a microscopic derivation of urban scaling relations for economic quantities vs. population, obtained from the consideration of social and infrastructural properties common to all cities, implies an effective model of economic output in the form of a Cobb-Douglas type production function. As a result we derive a new expression for the Total Factor Productivity (TFP) of urban areas, which is the standard measure of economic productivity per unit of aggregate production factors (labor and capital). Using these results we empirically demonstrate that there is a systematic dependence of urban productivity on city population size, resulting from the mismatch between the size dependence of wages and labor, so that in contemporary US cities productivity increases by about 11% with each doubling of their population. Moreover, deviations from the average scale dependence of economic output, capturing the effect of local factors, including history and other local contingencies, also manifest surprising regularities. Although, productivity is maximized by the combination of high wages and low labor input, high productivity cities show invariably high wages and high levels of employment relative to their size expectation. Conversely, low productivity cities show both low wages and employment. These results shed new light on the microscopic processes that underlie urban economic productivity, explain the emergence of effective aggregate urban economic output models in terms of labor and capital inputs and may inform the development of economic theory related to growth.

## Introduction

The importance of population size as a major determinant of the intensity of socio-economic activity in urban areas has recently been emphasized by research applying scaling analyzes to a diverse spectrum of urban indicators [1], [2], [3], [4]. Scaling analysis, which quantifies how measurable aggregate characteristics respond to a change in the size of the system, has been a powerful tool across a broad spectrum of science and technology research. Its analytical punch stems from the observation that this response is often a simple, regular, and systematic function over a wide range of sizes, indicating that there are underlying generic constraints at work on the system as it develops.

Cities, too, manifest non-trivial scaling across many metrics, both infrastructural and socio-economic, and scale in a similar way across a variety of urban systems worldwide. This is surprising since cities are quintessential complex adaptive systems manifesting multiple spatio-temporal scales with emergent dynamics that are typically viewed as historically contingent. Nevertheless, simple power law scaling is a good universal characterization of the average characteristic of cities world-wide, suggesting that a common organization and dynamics is at play in their development and economies, independent of local history, geography and culture [1], [3]. To be clear, we do not claim that there is a causal relation between urban scaling and urban productivity; scaling reveals a systematic relationship between urban population size and productivity, which itself is a manifestation of a more general relationship between population size and productivity [5], [6]. Causality stems from the ways in which being embedded inside larger agglomerations fundamentally affects how individuals interact with each other.

The scaling perspective, which may be familiar from the application of physics-based approaches to studying other complex systems, is reminiscent of another, seemingly unrelated, set of scaling relations that serve as the starting point for most economic approaches to cities and other economic units, such as firms or nations. The methodological hallmark of modern economics for discussing and quantifying the sources of economic growth and the determinants of productivity is a *production function*. Basically, a production function encapsulates a compact description of how aggregate economic output is generated from aggregate inputs, such as labor and capital. The conditions under which specific forms of a production function can be used to capture economic activity in cities within an urban system are often simply assumed and very rarely verified (see, for example, [7]). The major contribution of this paper is to address the question of how specific forms of production functions, common to all cities, emerge as effective models of economic output as a result of the observation of urban scaling relations and their theoretical underpinnings. We believe that the resulting synthesis, obtained from unifying these physics and economics-based perspectives, potentially leads to new and useful insights into the socio-economic dynamics of cities.

The derivation of specific forms of urban production functions also leads to a new analysis of the economic productivity of cities. Much research has been carried out over the past two decades on the causes of productivity differences across urban areas. The prevalent methodological approach has been to utilize a variant of the so-called *growth accounting method* [8] in order to statistically examine which of the myriad characteristics of urban areas affect their economic productivity [9], [10], [11], [12], [13], [14], [15], [16], [17]. This procedure relies on the assumption of a specific form of production function, such as Cobb-Douglas, and thereby on the identification of changes in its pre-factor, usually referred to as *total factor productivity*, as the fundamental measure of changes in economic productivity.

Agglomeration economies–a set of phenomena ultimately dependent on the size and density of urban populations–have been highlighted in the literature as causal mechanisms for the productivity-enhancing effects of scale and concentration in cities [18], [19], [20], [21], [22], [23], [24], [25]. An earlier literature documented the positive correlations between urban (population) size and productivity, measured as average wage or value added [26], [27], [28], [29]. The positive relationship between urban size and productivity is indeed a central fact of urban economics, and understanding its origins remains a major challenge in understanding cities. Thus, a derivation of a production function for cities that explains and constrains these analyzes would potentially make an important contribution to the understanding of the productivity of urban economies.

The paper is organized as follows. The next section briefly introduces and reviews scaling analysis. Section 2.2 builds upon the scaling relationship to construct scale-adjusted indicators of metropolitan performance. Section 2.3 derives the form of a general production function. Section 2.4 uses urban scaling relations to derive an analytical expression for the urban production function. Finally, section 2.5 shows our empirical estimation for the scale-adjusted productivity of U.S. urban areas and its statistical patterns. We close by presenting our conclusions and discussing the implications of the present results for further research.

## Results

### Scaling Analysis in Urban Systems

One fundamental aspect of cities is that most of their properties are not simply proportional to population size. For example larger cities tend to display larger per capita outputs in many their socio-economic quantities, from violent crime to wages, and need less material infrastructure per person (from roads to cables and pipes), though also use it more intensely [1]. These properties, and their detailed observed quantitative expression in terms of scaling relations can be derived from a microscopic theory that describes cities as co-located mixing social networks, subject to certain general efficiency constraints [5].

Specifically, scaling relations characterize how a given quantity of interest, *Y,* depends on a measure of the size of a system, *N*. A common feature of scaling is *scale invariance*, which corresponds to a relationship formalized as:
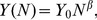
(1)where 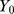 is a normalization constant and β is the scaling exponent (which can also be interpreted as an elasticity, as usually defined in economics). The significance of this power-law relation becomes clear when we consider an arbitrary scale change by a factor *λ* from *N* to *λN*. This induces a change in *Y* from *Y(N)* to *Y(λN)* that, without loss of generality, can be expressed as



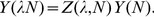
(2)When the scale factor *Z* depends only on λ, i.e. 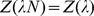, equation (2) can be solved uniquely to give the scale-invariant result of equation (1), with 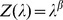. Scale-invariance implies that such a relationship–the ratio 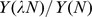
–is parameterized by a single dimensionless number, *β*. The ratio 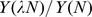 is independent of the particular system size *N* but is dependent on the ratio between sizes, λ; such systems are often referred to as self-similar [30]. Non-interacting systems, e.g. an ideal gas, are strictly extensive and are characterized by *β*  = 1. Most complex systems that can exist over a range of scales, from river networks to organisms, and from cities to ecosystems, are characterized typically by *β* different from unity; with open ended complex system typically displaying productivity that is superlinear, *β* >1.

Equation (1) bears a close resemblance to a *production function* (discussed in detail below), with *Y* denoting total economic output and *N* the size of urban population or labor pools (see, e.g., [14]). On a *per capita* basis, Equation (1) implies 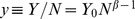, which can be interpreted, for example, as an equation for output per person as a function of the maximal number of people sharing ideas with each other [31]. In this sense the mathematical expression of economic output in terms of production functions and scaling analysis of general complex systems are very similar, although superficially originating from different perspectives. Below we show explicitly how these two pictures are related by deriving the form of the urban production function from scaling relations and their underlying microscopic dynamics.

### Scale-Adjusted Metropolitan Indicators

Scaling relations and production functions express only average expectations for (economic) outputs in terms of sets of inputs. But, as has been recently shown [32], the correct statistical interpretation of scaling laws is as expectation values for the quantity *Y,* conditional on the population size of a city; that is the mean associated with the probability density *P(Y|N).*

**Figure 1:**
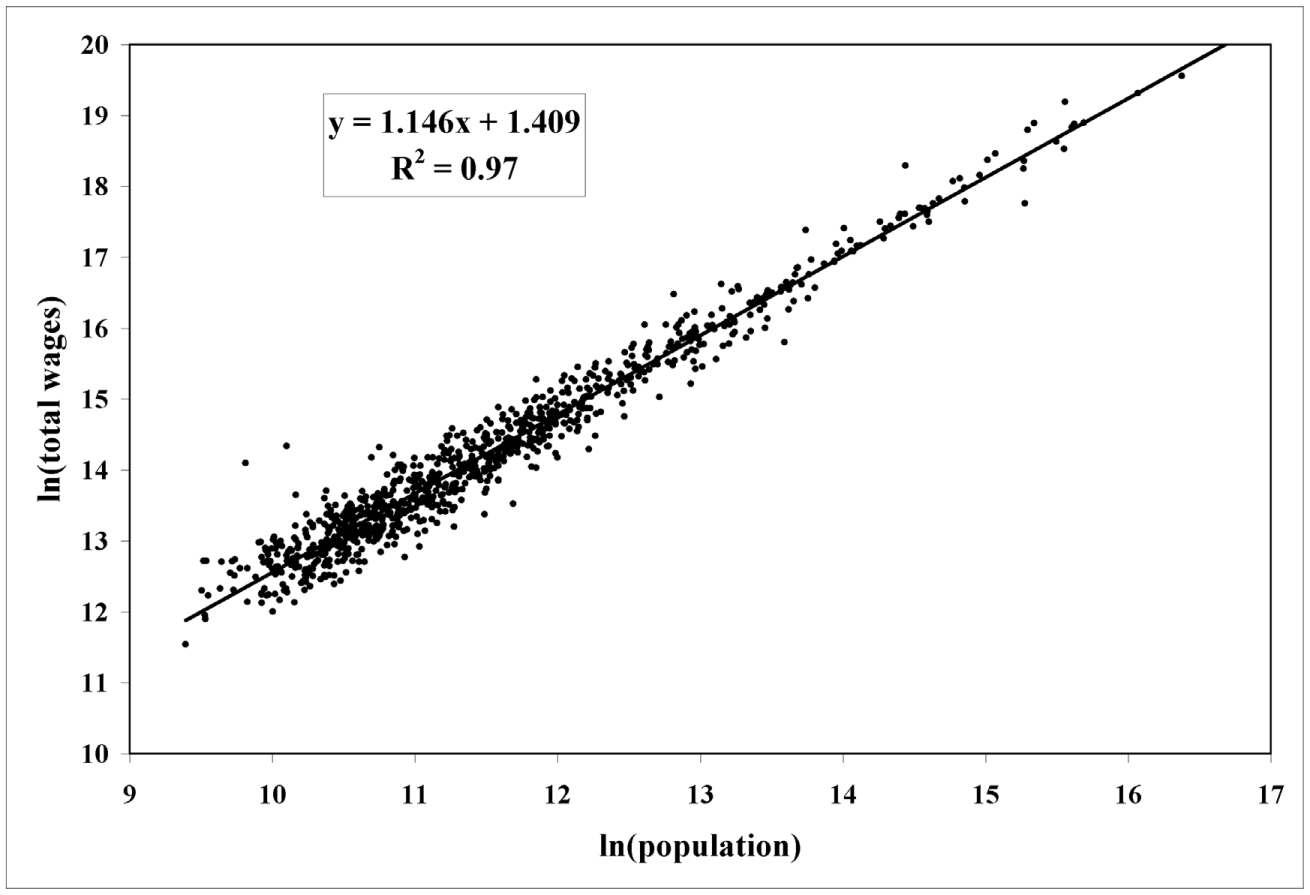
Scaling of total wages using data for all 943 urban areas of the United States (smoothed over the 2009–2011 period) showing superlinear scaling.

**Figure 2:**
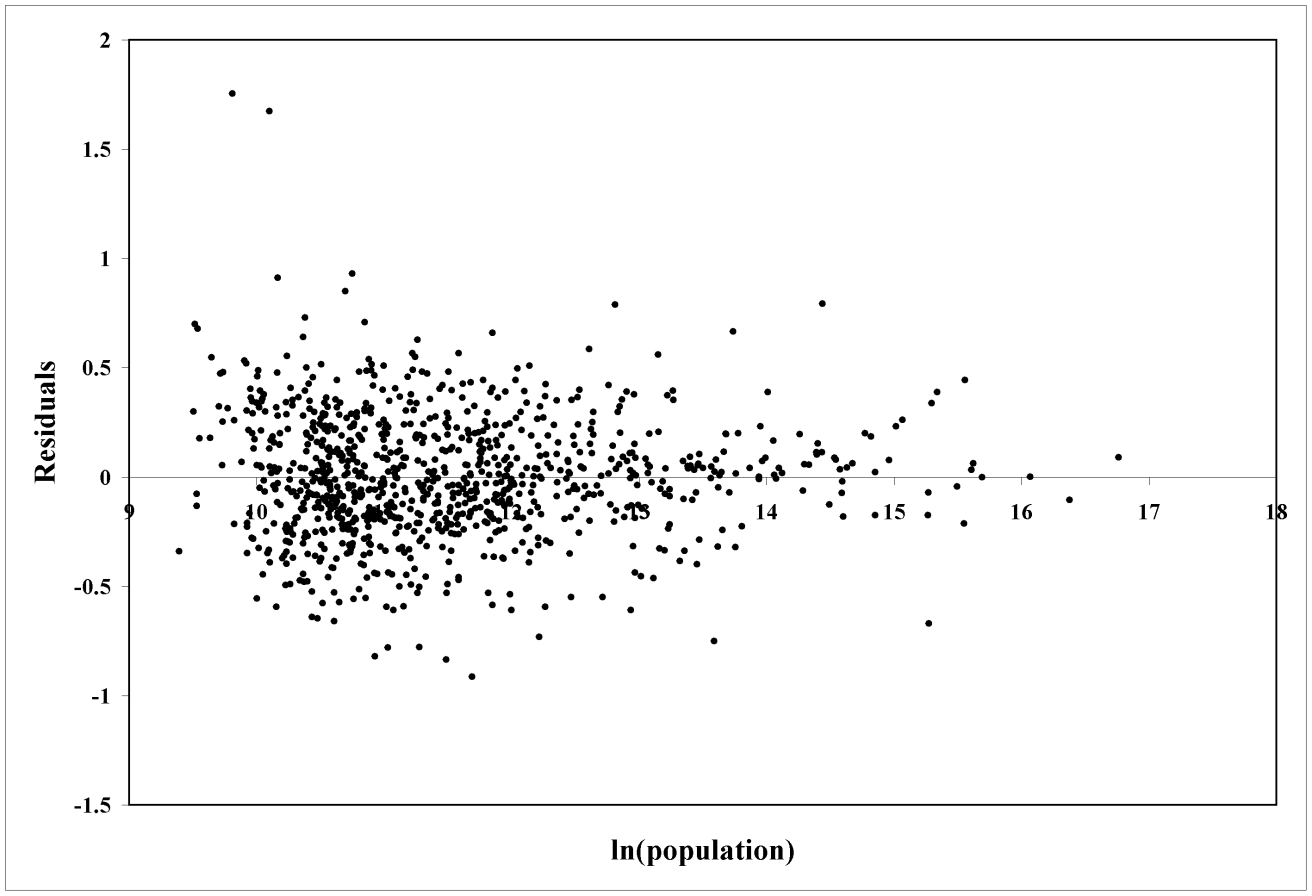
Residuals from regressing *ln(total wages)* on *ln(population)* using data for all 943 urban areas of the United States smoothed over the 2009–2011 period.

The statistical fluctuations about the mean scaling law, together with the value of the scaling parameters, can be determined using the log-transformed version of equation (1):
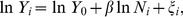
(3)with urban areas indexed by *i*. Here, the fluctuations or “random shocks” 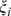 represent local (city-specific) deviations from the scale-invariant form. As an example of an urban metric that exhibits scaling behavior consider total wages, defined as the sum total of wages and salaries earned by residents in an urban area. Ordinary least squares estimation (OLS) of equation (3)– correcting for heteroskedasticity, and using data on *Total Wages* (*TW*) and population for the 943 urban areas of the United States (which consists of 367 Metropolitan Statistical Areas (MSAs) and 576 Micropolitan Areas, see Materials and Methods) smoothed over the 2009–2011 period–gives the following result:
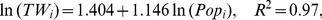
(4)with p-values virtually zero. Figure 1 shows the scatter plot of the data and the fitted regression line; a plot (Figure 2) clearly shows that they are scale-independent. Thus, a 1% increase in population is associated on average with a 1.15% increase in output, regardless of city size, in general agreement with theoretical expectations for β∼7/6 [5]. These self-similar and increasing returns to scale establish quantitatively the economic advantages of large cities (for further evidence of scaling behavior regarding urban characteristics see [1], [2], [3], [4]).

Equation (4) expresses the average productivity for a city of size *N*. Deviations from this average behavior capture the characteristics of each individual urban area not accounted for by the general agglomeration effects of population size. These deviations can be quantified by writing the residual equation in (3) as
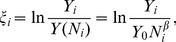
(5)where *Y_i_* is the observed value of output for each metropolitan area. We refer to *ξ* as a *Scale-Adjusted Metropolitan Indicator* (SAMI) [3]. The construction of SAMIs is similar to other uses of the method of residues [33]. Unlike per capita indicators, SAMIs are dimensionless and, by construction, independent of urban size [34]. SAMIs can be constructed for any variable capturing features of urban life which are subject to scaling agglomeration effects. (The deviations from the fitted line in Figure 1 and the residuals plotted in Figure 2 are in effect the SAMIs for total wages.) As a result of these definitions we can write any stochastic urban indicator, exactly, as



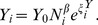
(6)We are now ready to derive the economic production function of cities from their probabilistic scaling properties.

### General Derivation of Economic Production Functions

We briefly recapitulate the derivation of a general production function in order to set up the theoretical framework. We proceed by first stating (as in [35]) an accounting relation: at any time, *t*,
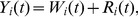
(7)with *Y* signifying the pecuniary value of the total output generated in the *ith* metropolitan area, *W* denoting its total labor income, and *R* its total capital income. It is from the observables in equation (7) that a putative production function is built. The production factor shares are defined as:



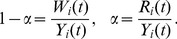
(8)Note that in general 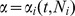 is city specific and a function of both time and population size *N*. We can differentiate equation (7) with respect to time (or with respect to *N*) and divide by output, *Y*, to obtain
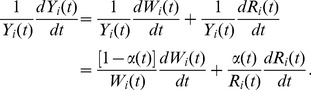
(9)


This can be integrated to give
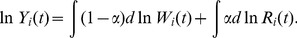
(10)


Integration by parts then yields the general result:
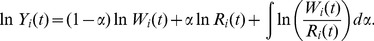
(11)


The last integral can be written as
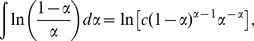
(12)where *c* is a constant of integration, so that, finally,



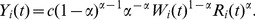
(13)We note that equation (13) is an instantiation of a more general relationship (for arbitrary γ), which can in turn be derived algebraically (note too that for the free factor to be independent of the production factors α must be a constant). We prefer the derivation presented here so as to highlight that *Y*, *W* and *R* are functions of time.

Constraining the solution in equation (11) to be consistent with the original equation (7) determines *c* = 1. This solution is general in that it does not require, for example, that the factor shares, α, be constant in time or population size. Thus the derivation of a Cobb-Douglas type production function (see also below) follows directly from the definitions (7–8) and does not carry more specific economic significance beyond that contained in these relations. In fact, and not withstanding its prominent role in the history of economic analyses, the Cobb-Douglas production function is basically a trivial identity that follows from a simple dimensional argument: since *Y*, *W* and *R* must have the same dimensions, and assuming that *Y* is solely composed of *W* and *R*, it must be expressible as equation (13), with exponents adding up to unity. However, this formalism takes on potentially greater usefulness when α is, in fact, a constant, independent of both time and population size; that is, when
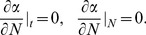
(14)


**Figure 3:**
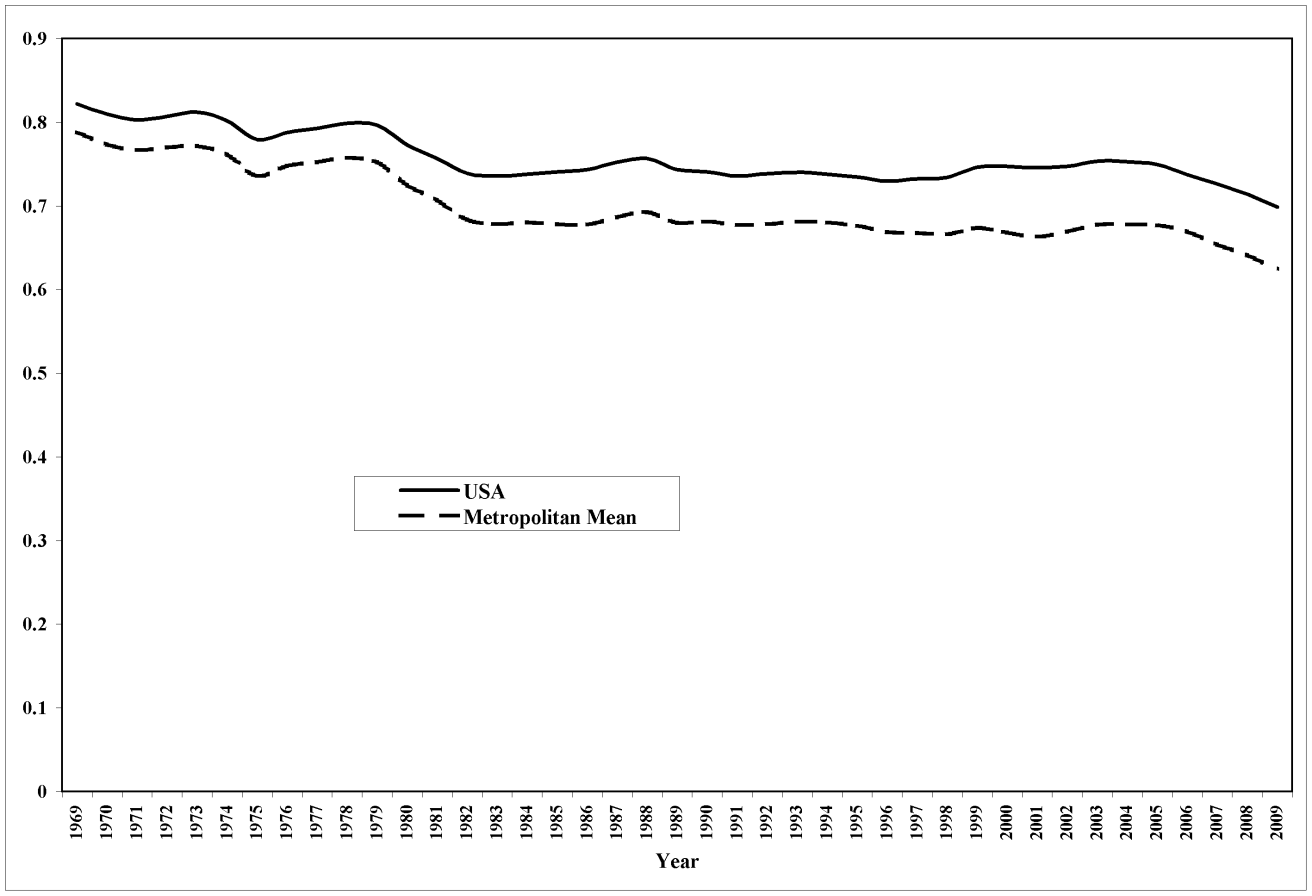
Ratio of urban labor income to total income (*1– α*) for MSAs and Micropolitan Areas in the U.S. 1969–2009.

Although the constancy of α is typically assumed when using production functions, its validity at the urban level is rarely confronted by data. We have performed an analysis using data for U.S. urban areas to check its empirical basis. The share of total income accruing to labor, *1– α,* can be calculated for both Metropolitan Statistical Areas (MSAs) and Micropolitan Statistical Areas, which together constitute the entire urban system of the United States. Figure 3 shows the time series, from 1969 to 2009, for the economy-wide value and the urban mean of *1– α*. Urban labor’s share of total income displays roughly the same temporal trend as the national labor’s share of income, both hovering around a value of 0.70 (the coefficient of variation for *1– α* is approximately 0.15 within each year). The correlation between the values of (*1– α)* specific to urban areas and their population size hovers around a paltry 0.05 over the whole of the period for which we have data: the share of total urban income accruing to location-specific labor is not a function of urban population size. (There is evidence that labor’s share of total national income is declining in the U.S. although the argument presented here holds even if this is the case. For a review of the evidence go to www.clevelandfed.org/research/trends/2012/0212/01gropro.cfm).

Equation (11), under the assumption of constant α, can be easily related to the familiar Cobb-Douglas production function, which is a widely used model for national and urban economies (see, for example, [7], [13], [14], [36]). This requires the introduction of conversion factors relating wages, *W_i_*(*t*), to labor input, *L_i_*(*t*), and capital income, *R_i_*(*t*), to capital input, *K_i_*(*t*) :):
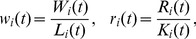
(15)
*w* is average wage, while *r* is the average rental price of capital. We can then write, *Y*, in the more familiar form
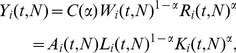
(16)with 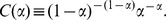 The pre-factor A(t,N) is often referred to as the total factor productivity (TFP) of the ith urban area and is the preferred measure of its economic productivity. A larger or smaller TFP multiplies the same factor inputs of labor and capital to produce greater or smaller economic output, respectively. Thus, the value of the TFP is interpreted as a body of technologies that allow the same input factors to produce a more valuable output, for example by shifting labor and capital from “basic” agriculture to “high-tech” industries. Technology, as captured by the value of A, should be interpreted broadly so that it can encompass all the social, demographic, technological, environmental, policy and even cultural factors that determine the overall productivity of an urban area. Finally, from equation (16) we obtain the following expression for urban TFP as a function of the productivity of labor and capital:



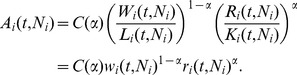
(17)Next, we show how the existence of scaling relations determines the form of *A*, resulting in its systematic parameterization as an explicit function of population size, *N*_i_, and specific local deviations, 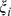.

### Derivation of Urban Total Factor Productivity From Scaling

So far we have explored the consequences of an accounting relation, equation (7) and the definition of factor shares, equation (8), together with the conservation laws expressed in equation (14), to obtain a Cobb-Douglas type production function common to all cities. We now show that the constancy of *α* is a consequence of urban scaling relations and their underlying microscopic dynamics, and use these relations to obtain a new expression for *A*_i_(t,*N*_i_). First note that, with
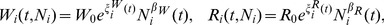
(18)it follows that



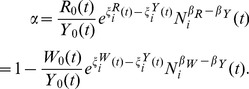
(19)Thus, for α to be independent of *N* is equivalent to requiring that both wages and rents scale with the same exponent, so that 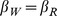. This is predicted from theory [5] as both quantities result from socioeconomic interactions in the city, and, as we showed above, empirically observed for U.S. cities, as 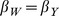, within their statistical confidence intervals. Consequently, the observation of *universal* socioeconomic superlinear urban scaling and its theoretical underpinnings imply the conservation of α vs. *N* and a Cobb-Douglas general form for the economic output of cities vs. population size.

The constancy of α in time is more problematic as it requires that the pre-factors *W_0_* and *R_0_* share the same time dependence, and that the differences between the SAMIs for location-specific total wages and total capital income, and the SAMI for total output also be time independent. The former relate to urban system-wide (national) economic growth and as such can be expected to vary slowly in time. The latter do change slowly in time [3], but analysis of their statistics reveals that their variance (recall that the SAMIs have zero mean) is approximately time independent [3], [32], as such we can expect that the average of α over the SAMIs is also approximately time independent. The deeper reasons for the approximate time independence of these quantities remain an important open problem grounded on the theory of economic growth, beyond the scope of the present paper.

Assuming the constancy of α from the previous arguments, we now derive an explicit expression for the TFP of cities. We first note that both the numerator and denominator in the expressions for wage per worker and average capital rent exhibit scaling behavior so that the marginal productivity of the two production factors can be recast using their associated SAMIs as:
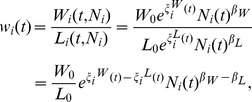
(20)

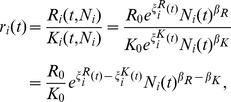
(21)


The term for TFP then takes the general form:
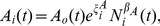
(22)with



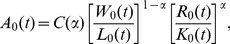
(23)

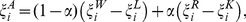
(24)

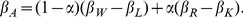
(25)


Equations (22)-(25) make explicit how urban TFP depends on both population size, through the scaling exponents, and on local, scale-independent fluctuations through the SAMIs. Equation (22) differs from a standard TFP formulation in that the productivity-enhancing effects of population are explicitly controlled for and the population-neutral effects explicitly represented by the term in equation (24). As a consequence any additional urban property proposed to explain a higher or lower productivity of specific cities not tied to their size (see below) must be expressed in terms of its contribution to the SAMIs for *W, L, R* and *K*.

Evaluating *A* requires knowledge of how *K*, the metropolitan capital stock, scales with urban size. Unfortunately, reliable data on urban capital stocks in the U.S. are not available at present. We can, however, estimate the value of the scaling coefficient for urban TFP by making a set of standard arguments. Given the observed values for the scaling coefficients for total wages and labor, *β_W_* ≈ 1.15 and *β_L_* ≈ 1, and with (1−*α*)_≈0.7_ the first term to the right of the equal sign on equation (24) has a value of 0.11 What about the value of the 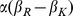 term? Under the widely-made assumption [34] that the rental price of capital. *r*, is constant, or nearly so, across metropolitan areas, and given that 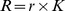 or equivalently, 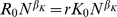 then 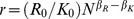 For *r* to be a constant, we must have 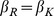. Therefore, *β_A_* ≈ 0.11 implying that urban productivity, measured by the TFP, increases on average by about 11% with each doubling of population.

The systematic (*i.e*., average) dependence of *A* on urban population size thus originates in the mismatches of the scaling of total wages. *W*, versus labor, *L*, and, potentially, of capital income, *R*, versus capital returns, *K*. Given the observed values for the scaling coefficients for total wages and labor, their difference can generate an average increase in productivity resulting from a self-similar wage premium for the same amount of labor (and also, potentially, a savings in the amount of labor input). The scale-adjusted measure for urban TFP can be well-approximated by:
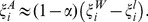
(26)


Below we measure these quantities in order to shed light on the ways in which cities can be more or less economically productive independently of their population size.

### Decomposition of Urban Total Factor Productivity

We calculated the scale-adjusted TFP using equation (26) and data for both Metropolitan and Micropolitan Areas averaged over the period 2001–2005, and setting *1– α* (labor’s share of income), to be 0.7. For this decomposition we only use data on metropolitan wages and employment as these two variables are directly and unambiguously measurable.

The top fifty urban areas, ranked according to the values of their scale-adjusted productivity, *ξ^A^*, are shown on Table 1, while Table 2 shows the rankings for the top fifty Metropolitan Areas (MSAs). One result immediately stands out: the absence of most of the large metropolitan areas from the top ranks of the most productive urban centers in contrast to a ranking generated by simply using the conventional output per worker as the measure of productivity. The scale-adjusted measure of urban TFP removes the productivity-enhancing effects of population size thereby identifying the truly most productive urban areas–the standard ranking using per capita measures seriously overestimates the largest metropolitan areas’ productivity.

**Table 1:** Top 50 urban areas, ranked by their scale-adjusted measure of TFP (*ξ^A^*).

	Urban Area	*ξ^A^*	*ξ^W^*	*ξ^L^*
1	Los Alamos, NM (Micropolitan Area)	0.6964	1.7771	0.7822
2	San Jose-Sunnyvale-Santa Clara, CA (Metropolitan Area)	0.3674	0.6155	0.0907
3	Gillette, WY (Micropolitan Area)	0.3480	0.7895	0.2923
4	Bridgeport-Stamford-Norwalk, CT (Metropolitan Area)	0.3342	0.5672	0.0898
5	Rock Springs, WY (Micropolitan Area)	0.2937	0.6664	0.2467
6	Trenton-Ewing, NJ (Metropolitan Area)	0.2799	0.6054	0.2056
7	Harriman, TN (Micropolitan Area)	0.2791	0.1053	−0.2934
8	Midland, MI (Micropolitan Area)	0.2691	0.3906	0.0061
9	Kokomo, IN (Metropolitan Area)	0.2652	0.4415	0.0627
10	Elko, NV (Micropolitan Area)	0.2544	0.4585	0.0950
11	Sidney, OH (Micropolitan Area)	0.2369	0.6268	0.2884
12	Borger, TX (Micropolitan Area)	0.2328	0.2749	−0.0576
13	Marshfield-Wisconsin Rapids, WI (Micropolitan Area)	0.2196	0.5390	0.2253
14	Lexington Park, MD (Micropolitan Area)	0.2189	0.3729	0.0602
15	Wilmington, OH (Micropolitan Area)	0.2045	0.5831	0.2909
16	Columbus, IN (Metropolitan Area)	0.1995	0.5330	0.2480
17	Connersville, IN (Micropolitan Area)	0.1845	0.1965	−0.0671
18	Columbia, TN (Micropolitan Area)	0.1783	0.3424	0.0878
19	Boulder, CO (Metropolitan Area)	0.1776	0.5536	0.3000
20	Hinesville-Fort Stewart, GA (Metropolitan Area)	0.1762	0.1730	−0.0787
21	Oshkosh-Neenah, WI (Metropolitan Area)	0.1731	0.4166	0.1694
22	Ann Arbor, MI (Metropolitan Area)	0.1728	0.4689	0.2220
23	Durham-Chapel Hill, NC (Metropolitan Area)	0.1715	0.4795	0.2344
24	Bellefontaine, OH (Micropolitan Area)	0.1676	0.2733	0.0340
25	Auburn, IN (Micropolitan Area)	0.1652	0.4951	0.2590
26	Bloomington-Normal, IL (Metropolitan Area)	0.1643	0.4435	0.2089
27	Defiance, OH (Micropolitan Area)	0.1640	0.3351	0.1008
28	Corning, NY (Micropolitan Area)	0.1636	0.1331	−0.1006
29	Battle Creek, MI (Metropolitan Area)	0.1612	0.1723	−0.0579
30	Andrews, TX (Micropolitan Area)	0.1559	0.1135	−0.1092
31	Pahrump, NV (Micropolitan Area)	0.1546	−0.0364	−0.2573
32	Fort Leonard Wood, MO (Micropolitan Area)	0.1542	0.2880	0.0677
33	Carson City, NV (Metropolitan Area)	0.1540	0.5265	0.3065
34	Norwich-New London, CT (Metropolitan Area)	0.1534	0.3287	0.1095
35	Decatur, IL (Metropolitan Area)	0.1533	0.2927	0.0736
36	St. Marys, GA (Micropolitan Area)	0.1511	0.1630	−0.0529
37	Rochester, MN (Metropolitan Area)	0.1511	0.4771	0.2613
38	Warsaw, IN (Micropolitan Area)	0.1510	0.2754	0.0597
39	Manchester-Nashua, NH (Metropolitan Area)	0.1471	0.2958	0.0857
40	Wilson, NC (Micropolitan Area)	0.1450	0.2973	0.0902
41	Fort Valley, GA (Micropolitan Area)	0.1395	−0.0795	−0.2787
42	Hartford, CT (Metropolitan Area)	0.1357	0.2802	0.0864
43	Crawfordsville, IN (Micropolitan Area)	0.1351	0.2644	0.0714
44	LaGrange, GA (Micropolitan Area)	0.1321	0.3561	0.1674
45	Owatonna, MN (Micropolitan Area)	0.1316	0.4748	0.2869
46	Warner Robins, GA (Metropolitan Area)	0.1313	0.2055	0.0178
47	Findlay, OH (Micropolitan Area)	0.1304	0.4602	0.2739
48	Racine, WI (Metropolitan Area)	0.1285	0.0224	−0.1612
49	Kennewick-Pasco-Richland, WA (Metropolitan Area)	0.1281	0.1230	−0.0600
50	San Francisco-Oakland-Fremont, CA (Metropolitan Area)	0.1241	0.2166	0.0394

**Table 2:** Top 50 metropolitan areas, ranked by their scale-adjusted TFP (*ξ^A^*).

	Area	*ξ^A^*	*ξ^W^*	*ξ^L^*
1	San Jose-Sunnyvale-Santa Clara, CA	0.4743	0.7609	0.0834
2	Bridgeport-Stamford-Norwalk, CT	0.4433	0.7178	0.0845
3	Trenton-Ewing, NJ	0.3917	0.7567	0.1972
4	Kokomo, IN	0.3784	0.5597	0.0192
5	Columbus, IN	0.3140	0.6575	0.2088
6	Hinesville-Fort Stewart, GA	0.2920	0.3145	−0.1026
7	Oshkosh-Neenah, WI	0.2860	0.5504	0.1418
8	Ann Arbor, MI	0.2856	0.6314	0.2235
9	Boulder, CO	0.2852	0.6337	0.2263
10	Durham-Chapel Hill, NC	0.2839	0.6480	0.2424
11	Bloomington-Normal, IL	0.2789	0.6014	0.2030
12	Battle Creek, MI	0.2742	0.3022	−0.0895
13	Carson City, NV	0.2709	0.6742	0.2871
14	Norwich-New London, CT	0.2659	0.4771	0.0973
15	Rochester, MN	0.2657	0.6396	0.2599
16	Decatur, IL	0.2652	0.3952	0.0164
17	Manchester-Nashua, NH	0.2588	0.4495	0.0798
18	Warner Robins, GA	0.2489	0.3947	0.0392
19	Hartford-West Hartford-East Hartford, CT	0.2449	0.4428	0.0930
20	Kennewick-Pasco-Richland, WA	0.2445	0.3191	−0.0303
21	Racine, WI	0.2420	0.1725	−0.1733
22	Huntsville, AL	0.2343	0.4667	0.1320
23	Vineland-Millville-Bridgeton, NJ	0.2321	0.1744	−0.1572
24	San Francisco-Oakland-Fremont, CA	0.2292	0.3744	0.0469
25	Napa, CA	0.2287	0.5025	0.1757
26	Ithaca, NY	0.2151	0.4459	0.1386
27	Washington-Arlington-Alexandria, DC-VA-MD-WV	0.2146	0.4588	0.1522
28	Monroe, MI	0.2146	−0.0639	−0.3705
29	Saginaw-Saginaw Township North, MI	0.2130	0.2330	−0.0712
30	Longview, WA	0.2101	0.1487	−0.1515
31	Springfield, IL	0.2081	0.4361	0.1389
32	Sheboygan, WI	0.2079	0.4470	0.1500
33	Atlantic City-Hammonton, NJ	0.2050	0.4705	0.1776
34	Dalton, GA	0.2048	0.4995	0.2069
35	Boston-Cambridge-Quincy, MA-NH	0.1980	0.3698	0.0870
36	Sandusky, OH	0.1974	0.3751	0.0931
37	Elkhart-Goshen, IN	0.1925	0.5796	0.3046
38	Janesville, WI	0.1899	0.2121	−0.0591
39	Corvallis, OR	0.1840	0.4342	0.1713
40	Burlington-South Burlington, VT	0.1837	0.4833	0.2209
41	Mansfield, OH	0.1828	0.2294	−0.0318
42	Peoria, IL	0.1783	0.2633	0.0086
43	Rome, GA	0.1781	0.2529	−0.0015
44	New Haven-Milford, CT	0.1779	0.2168	−0.0374
45	Holland-Grand Haven, MI	0.1757	0.2310	−0.0201
46	Cheyenne, WY	0.1749	0.4234	0.1736
47	Cedar Rapids, IA	0.1722	0.3898	0.1438
48	Spartanburg, SC	0.1717	0.2269	−0.0183
49	Harrisburg-Carlisle, PA	0.1716	0.4782	0.2330
50	Bay City, MI	0.1657	0.0233	−0.2134

**Figure 4:**
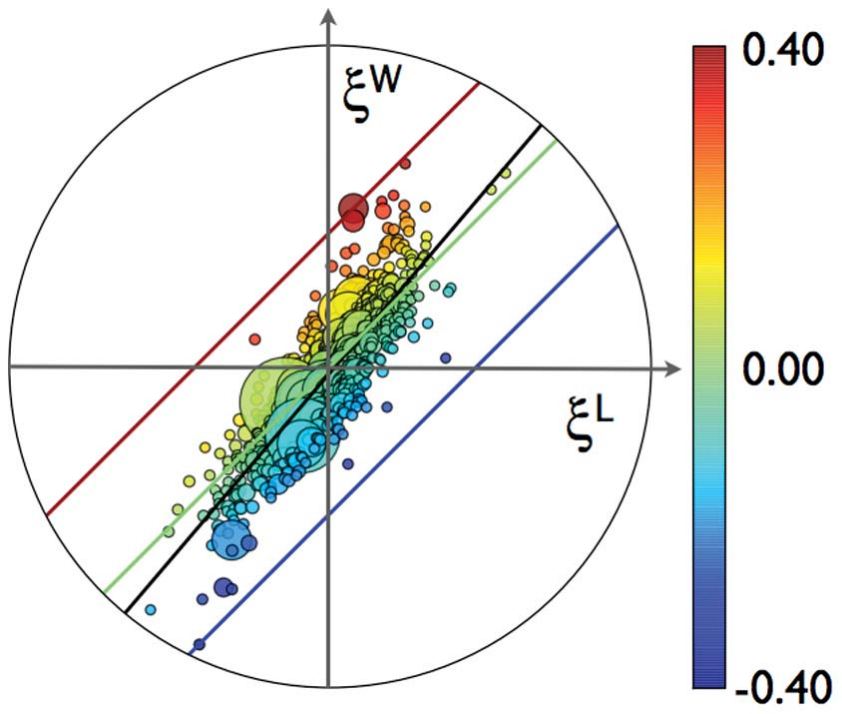
The SAMIs for urban areas’ TFP (color) in the *ξ^W^- ξ^L^* plane. The size of each symbol denotes its population (smallest cities are shown at the same small symbol size). The solid green line divides the space into TFPs above (positive) and below (negative) the expected value for each city’s population. The solid red line is the equal TPF parameter space for Silicon Valley, while the solid blue line is the equal TFP space for the least productive city in the sample (Rio Grande City-Roma, TX). The black solid line shows the linear best fit to the data *ξ^W^* = −0.02+1.17 *ξ^L^* (R^2^ = 0.74).

Figure 4 shows all urban areas in terms of their two performance metrics: the SAMIs for wages, *ξ^W^*, and labor, *ξ^L^*. The population size of each city is denoted by the size of the circles, and their scale adjusted productivity *ξ^A^* as their color. We easily see that the 45° solid green line divides the plane into two regions: above the line, where *ξ^A^* >0, urban areas display above average TFP and are denoted in warm colors (green to red); below the line, where *ξ^A^* >0, and denoted in cold colors (green to dark blue) appear urban areas with below average TFP. Perhaps the most striking aspect of Figure 4 is how narrow that band of values is; remarkably there are almost no cities in the second and forth quadrants far from the origin.

The results show an interesting trend in the exceptionality of urban TFPs, once population size has been factored out. While the way to maximize TFP is to maximize the difference 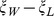; that is to have exceptionally high wages and exceptionally low labor input (employment), few cities with such properties exist (they would appear in the 2^nd^ quadrant of Figure 4). The urban area with the highest productivity, by far, is Los Alamos, the Micropolitan Area in New Mexico that hosted the Manhattan Project, not shown in Figure 3 because it is so far off-scale. Los Alamos, with a population of about 18,000 inhabitants, receives an annual investment of approximately $2.2 billon in federal funds allocated to Los Alamos National Laboratory. Los Alamos shows both exceptionally high wages and levels of employment, but clearly these are largely the result of a particular federal decision related to the high value of Los Alamos National Laboratory’s mission and its need for a small and remote location. The second highest urban TFP, even after accounting for population size, corresponds to Silicon Valley (the San Jose-Santa Clara, Metropolitan Area in California). San Jose also shows exceptionally high wages, and to a lesser extent high levels of employment. All other urban areas with highest TFP (dark red in Figure 4) share most of the same general characteristics. A singular exception is Harriman, TN, which shows a high TFP as a result of low levels of employment, and not particularly high wages.

To emphasize these points we show in Figure 4 several lines of equal TFP, which are parameterized by *ξ^W^* = C+*ξ^L^*, where the intercept *C = ξ^A^/α* is set for different values of *ξ^A^*. Τhe red solid line in Figure 4 maps the space of equal TFP at varying *ξ^W^* and *ξ^L^* for Silicon Valley. Note how no other urban area approaches the performance of San Jose, and no urban areas even come close among those with employment less than average (2^nd^ and 3^rd^ quadrants). Similarly the lowest possible TFP would correspond to low wages and high employment (4^th^ quadrant). The dark blue line, tracks the TFP of the lowest ranked metropolitan area: Rio Grande City-Roma, TX. Most actual cities with very low TFP, including the metropolitan areas of McAllen and Brownsville, TX, show similar patterns of low wages and low employment. However there are some exceptions, such as Vermillion (South Dakota), which shows exceptionally large employment (*ξ^L^* = 0.44) but only average total wages (*ξ^W = ^*0.03). While arguably these are signs of a functioning community it is penalized in terms of an exceptionally low TFP because its marginal product of labor (MPL) is small. A summary of these results is provided by a simple linear regression (*ξ^W^* = −0.02+1.17 *ξ^L^*, R^2^ = 0.74, black solid line), which is close to a 45-degree line but also shows a slightly greater slope emphasizing the trend for higher wages and lower employment in high TFP cities and lower wages and higher employment for those with lower TFP.

These results suggest that the principal objective of cities is not to maximize their productivity alone. In fact, as decentralized economies where economic optimization is driven primarily by individuals, the key property of economically successful cities may be to maximize wages and this in turn may lead to general high levels of employment through supporting activities. This close relationship between high wages and high levels of employment and vice-versa seems to be a general feature of urban economies in the U.S. It would be interesting to test it further in other nations, through time.

## Discussion

We have shown that an integrated consideration of the standard approach to urban areas as aggregate production devices and of the systematic dependence of the main factors of production on population size (via urban scaling) results in a specific form of a Cobb-Douglas type production function common to all cities. The resulting functional form manifests explicitly dependences of urban productivity on population size and local factors in terms of size-independent deviations (SAMIs). In particular, the analysis leads to a new expression for the total factor productivity (TFP) in terms of an explicit scale-invariant dependence on population size and on size-independent deviations due to the mismatch between labor income and employment (as well as capital income and capital stock).

We believe that these results provide some reassurance to urban economic theory, but, more importantly, a set of tight quantitative constraints that any model that aspires to describe real cities should satisfy. In fact, the decomposition of urban productivity through scaling analysis shows that the productivity of urban areas is actually a fairly low dimensional quantity characterized not only by a systematic average dependence on population size but also by a close relationship between exceptions to population size expectations in terms of wages and labor. This decomposition parallels, and may motivate, a re-examination of the sometimes difficult distinction between general urbanization effects common to all cities, which must be average functions of city size, and more particular localization effects that may be specific to a single city or to groups of cities.

It is the fact that larger deviations in magnitude occur for wages than for employment that makes this co-variation positive or negative. These results suggest that the economies of cities are not maximizing total productivity per se, as might be the case for a firm, but instead at providing environments for economic development and productivity enhancements that, when successful, lead to growth in both wages and employment. We believe that economic theory aimed at explaining the aggregate productivity of urban areas (in the U.S., at least) should be aimed at these clear and regular empirical relationships. It remains an open question for further study whether these relations apply to other urban systems, and to what extent the approximate time independence of the factors share, *α*, can be derived from a deeper understanding of the processes of economic growth at the regional and national levels.

## Materials and Methods

### Functional City Definitions

*Metropolitan* and *Micropolitan Statistical Areas* are defined by the U.S. Office of Management and Budget and are standardized county-based regions having at least one urbanized area (with 50,000 or more population in the case of MSAs or at least 10,000, but less than 50,000, in the case of Micropolitan Areas), plus adjacent territory with a high degree of social and economic integration with the core as measured by commuting ties. Both MSAs and Micropolitan Areas are in effect unified labor markets that represent a wide variety of geographic, demographic and socio-economic characteristics. There are 366 MSAs and 576 Micropolitan Areas in the USA as of June 2011.

### Data Sources

Data on Gross Metropolitan Product and on metropolitan employment, population and personal income are provided by the U.S. Commerce Department’s Bureau of Economic Analysis (BEA) (www.bea.gov/regional/index.htm#gsp). Total *personal income* is calculated as the sum of wage and salary disbursements, supplements to wages and salaries, proprietors’ income, rental, dividend and interest income, and personal current transfer receipts, less contributions for government social insurance, while *labor income* is the sum of wage and salary disbursements and supplements to wages and salaries. Data on total wages, employment and population were obtained from the Regional Economic Accounts also produced by the BEA (www.bea.gov/regional/reis/). Wage data was deflated using the Federal Reserve’s chain-type price index and is expressed in 2005 dollars (www.research.stlouisfed.org).
